# Challenges and opportunities in the development of metal-based anticancer theranostic agents

**DOI:** 10.1042/BSR20212160

**Published:** 2022-05-13

**Authors:** Shreyas P. Vaidya, Shubhankar Gadre, Ravi Teja Kamisetti, Malay Patra

**Affiliations:** Medicinal Chemistry and Cell Biology Laboratory, Department of Chemical Sciences, Tata Institute of Fundamental Research, Homi Bhabha Road, Mumbai, Maharashtra 400005, India

**Keywords:** Chemotherapy, diagnostics, Imaging, Medicinal Inorganic Chemistry, Platinum drugs, Theranostics

## Abstract

Around 10 million fatalities were recorded worldwide in 2020 due to cancer and statistical projections estimate the number to increase by 60% in 2040. With such a substantial rise in the global cancer burden, the disease will continue to impose a huge socio-economic burden on society. Currently, the most widely used clinical treatment modality is cytotoxic chemotherapy using platinum drugs which is used to treat variety of cancers. Despite its clinical success, critical challenges like resistance, off-target side effects and cancer variability often reduce its overall therapeutic efficiency. These challenges require faster diagnosis, simultaneous therapy and a more personalized approach toward cancer management. To this end, small-molecule ‘theranostic’ agents have presented a viable solution combining diagnosis and therapy into a single platform. In this review, we present a summary of recent efforts in the design and optimization of metal-based small-molecule ‘theranostic’ anticancer agents. Importantly, we highlight the advantages of a theranostic candidate over the purely therapeutic or diagnostic agent in terms of evaluation of its biological properties.

## Introduction

Cancer is a noncommunicable disease which is characterized by the development of abnormal cells that divide uncontrollably and have the ability to spread throughout the human body. Despite significant advances in the understanding of cancer cell biology and anticancer drug development, cancer remains as one of the leading causes of morbidity and mortality worldwide. With 10 million worldwide deaths in 2020, it became the second leading cause of death after cardiovascular diseases [1]. As per the prediction, the number of people diagnosed with cancer will increase by 60% within next two decades [2]. With the rapid rise in global cancer burden, the disease continues to impose a huge socio-economic burden on society.

Traditionally, surgery, radiotherapy and chemotherapy are the most widely used treatment options for cancer. Additionally, some modern therapeutic modalities such as hormone-based therapy, stem cell therapy, immunotherapy are approved for clinical use in specific cases or are in advanced stage of clinical trials [3,4]. However, chemotherapy with cytotoxic small-molecule drugs remains the mainstay of cancer treatment [4–6]. Chemotherapeutic drugs are effective against a variety of cancers and can also be employed for the treatment of metastatic cancers. Small-molecule based chemotherapeutics also tend to be affordable as compared to some of the other treatment modalities. Doxorubicin, Gemcitabine and Bleomycin are some examples of small-molecule based chemotherapeutic drugs which are used clinically. The field of metal-based drugs for chemotherapy has blossomed over the past few decades. Platinum (Pt) drugs cisplatin, carboplatin and oxaliplatin are used as frontline chemotherapeutics for treatment of many different types of cancers. Approximately 50% of cancer patients who are receiving chemotherapy are being treated with Pt drugs [7]. Despite their phenomenal clinical success, these cytotoxic blockbuster drugs suffer from numerous deficiencies which hamper their therapeutic potential. Resistance is often encountered during the treatment cycle which renders the drug ineffective and finally leads to treatment failure. Nonspecific accumulation of drugs in healthy tissues or organs causes severe side-effects. Nephrotoxicity, myelosuppression and neurotoxicity are the commonly observed side-effects for platinum-based drugs. These limitations are specific to patient as well as cancer types and stages; therefore, it is extremely difficult to come up with a general strategy to tackle these issues [8]. Therefore, the ability to detect the resistance in an individual patient against a particular drug as early as possible offers the possibility of switching over to another and more effective drug or therapy. Moreover, monitoring the therapy outcome has an added advantage in terms of deciding the suitable dose in each cycle depending on the state of disease progression. Side effects are directly related to the dose and therefore can be controlled to some extent by adjusting the dose. Currently the efficacy of anticancer therapy is monitored either by using direct methods such as computed tomography (CT) and magnetic resonance imaging (MRI) or by an indirect method employing 2-[fluorine-18] fluoro-2-deoxy-d-glucose (^18^F-FDG) to quantify metabolic activity through positron emission tomography (PET) imaging [8]. However, these diagnostic techniques are time consuming and require expensive instrumentation and settings. Moreover, lack of selectivity and specificity does not allow precise and real-time monitoring of therapeutic efficacy [9–11]. For example, in some cases no changes in the ^18^F-FDG uptake was noticed before and after therapy despite the treatment being effective in terms of tumor size regression [12].

**Figure 1:**
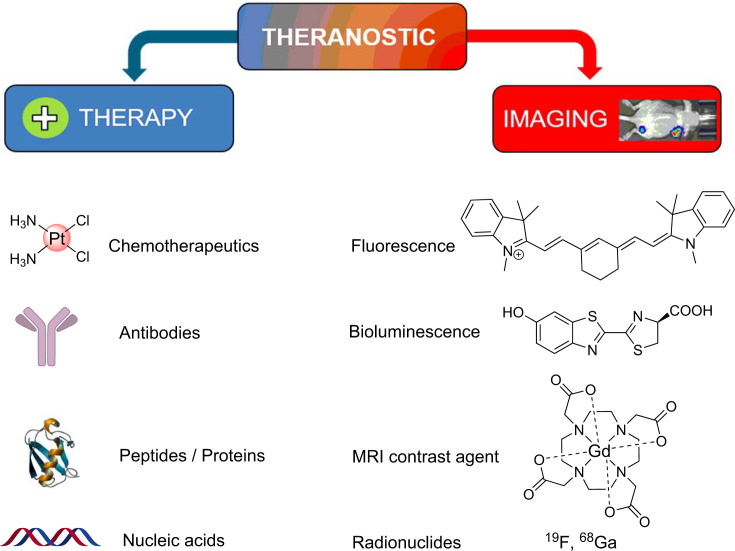
Schematic presentation of the concept of theranostic agent comprising of various individual treatment and imaging modalities

Therefore, smart materials or molecules with integrated therapeutic and diagnostic properties would be an ideal solution for efficient and safe cancer therapy. These materials are termed as ‘Theranostics’. The concept of theranostics which comprises of various individual treatment and diagnostic modalities is schematically presented in Figure 1 and reviewed elsewhere [13–20]. Tracing the origin of the term ‘Theranostic’ reveals that Funkhouser coined it in 2002 combining ‘therapy’ and ‘diagnosis’ to describe the business model of his company which involved in developing diagnostic tests for monitoring therapy [21]. Theranostic agents have numerous advantages over individual therapeutic and diagnostic candidates. Concurrently with therapeutic action, these materials are self-sufficient to report their accumulation in tumor and other body parts, enabling real-time noninvasive monitoring of the therapeutic progress and off-target accumulation. Therefore, they can be easily adopted for personalized cancer therapy. Moreover, owing to their real-time imaging competencies, studying in vivo pharmacokinetics and pharmacodynamics of theranostic candidates is relatively easy as compared to a therapeutic candidate in the developmental stage. Additionally, their cellular uptake mechanism, intracellular organelle localization, activation etc. can be evaluated directly using live cell imaging. The outcome of these assays are important determinants for assessing the clinical translatability of new metal-based drug candidates.

Recently, a great deal of attention has been devoted to the development of metal-based small molecule and nanoparticle-based theranostic agents. In this focused review, we will discuss the main developments in the field of small-molecule metal-based anticancer theranostics and theranostic conjugates using prominent examples. Along with proven clinical potential, metal complexes offer wide flexibility in terms of designing and functionalizing the molecules. This is possible due to their variable oxidation states, existence of radioactive isotopes, paramagnetism, and unique, tunable spectral properties. For the sake of clarity, we have categorized the theranostics on the basis of their diagnostic (imaging) aspects. Special emphasis has been given to the design principle of different classes of metal-based theranostics employing various imaging modalities such as optical, MRI, radioactive and infrared (IR). We have discussed about their in-depth mechanism of action and real-time trackability through imaging in living biological systems. Further we have assessed the advantages, disadvantages and future challenges associated with these classes of theranostic agents and suggested a few possible strategies, wherever possible, to improve their properties.

## Metal-based anticancer chemotherapeutic agents

**Figure 2:**
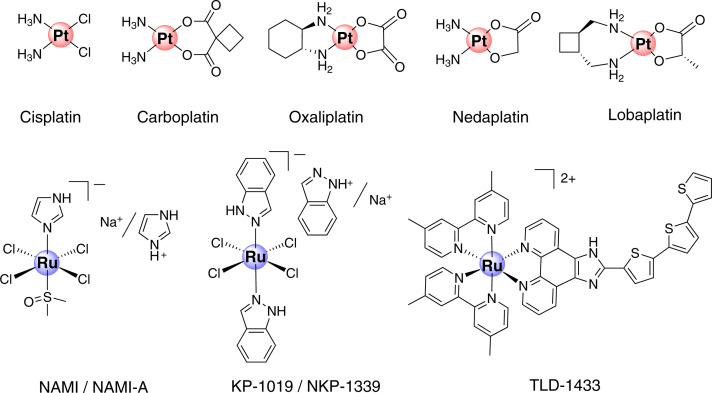
Clinically approved Pt-based anticancer drugs (top panel) and ruthenium-based anticancer compounds entered in clinical trials (bottom panel)

The usage of metals and metal-based compounds especially those of mercury, gold, silver, copper and arsenic for treatment of numerous diseases dates back to ancient times of recorded history. In the ancient times, metals were believed to possess near-miraculous power to eradicate most of the diseases. However, it was not until the beginning of 20th century that the potential of inorganic compounds as pharmaceuticals was realized and appreciated [22]. In 1910, Nobel laureate Paul Ehrlich demonstrated for the first time the application of inorganic chemistry in drug discovery [23,24]. Screening of more than thousands of organ-arsenic compounds led to the discovery of Ehrlich 606, named as Salvarsan. Salvarsan and its more water soluble but slightly less effective derivative Neosalvarsan were approved in 1910 and 1912, respectively, for the treatment of syphilis, an unpleasant and deadly disease caused by the spirochete *Treponema Pallidum* bacteria. It is worthy to note that the screening concept introduced by Paul Ehrlich is still one of the widely applied strategies in pharma industry as well as in academic settings for discovery of new leads or hits. Later, the accidental discovery of anticancer properties of Pt compound cisplatin, [(cis-Pt(NH_3_)_2_Cl_2_] by Rosenberg in 1965 and its subsequent FDA approval in 1978 for treatment of testicular, ovarian and bladder cancers ushered in the modern era of metal-based anticancer drugs (Figure 2) [25]. Since then, research on anticancer properties of metal complexes has gained increased attention. Two more Pt-based anticancer drugs carboplatin and oxaliplatin were approved in 1990 and 2002 for worldwide use. Moreover, nedaplatin and lobaplatin were approved for use in Japan and China, respectively (Figure 2). Many more Pt complexes are in preclinical and clinical development stage [26,27]. Despite being one of the most successful classes of anticancer agents in the clinic, Pt drugs suffer from a few serious drawbacks. Inherent as well as acquired resistance in cancer reduces the efficacy of Pt-based therapy. Moreover, severe side effects such as nephrotoxicity, neurotoxicity, myelosuppression affects the quality of life of patients undergoing Pt therapy [26]. To address these issues, anticancer potential of various non-Pt metal complexes of ruthenium (Ru), Gold (Au), Iridium (Ir), Rhenium (Re) etc. has been investigated [28–31]. So far, Ru has been demonstrated to be the most promising alternative to Pt [32,33]. In 1980s, Clarke et al*.* first demonstrated the potent anticancer activity of a Ru complex, fac-[Ru^III^Cl_3_(NH_3_)_3_] in EMT-6 sarcoma bearing mice [34,35]. Subsequently, Keppler et al*.* discovered imidazolium Ru^III^-based ionic compounds KP418 and KP1019/NKP-1339 which showed impressive anticancer efficacy in colorectal cancer mice model (Figure 2) [30]. NKP-1339 is currently being evaluated in clinical trial phase IIa [36]. Notably, NKP-1339 exerted efficacy in patients with cancers that were nonresponsive to other treatments. A subtle structural modification of the imidazolium Ru^III^ motif by Sava et al*.* led to the discovery of NAMI-A with antimetastatic activity, which also entered clinical trial phase I, but was later discontinued (Figure 2). Sadler et al*.* and Dyson et al*.* pioneered the discovery of two promising organometallic η^6^-π-bonded arene Ru^II^ anticancer complexes [27]. Prominent examples of this class include RM175 and RAPTA-C which are in advanced preclinical evaluation. Apart from that, various structurally inert polypyridyl complexes were also reported to show impressive antiproliferative activities [27]. Importantly, some of these compounds not only overcame Pt-resistance but also had a better safety and tolerability profile *in vivo*. Moreover, a ruthenium polypyridyl complex TLD-1433 entered clinical trial for photodynamic therapy of solid bladder cancer (Figure 2) [32]. Anticancer Au, Re, Ir based compounds are in early developmental stage. Despite excellent anticancer activities of some candidates, they are yet to be critically evaluated for their in-depth mechanism of action, pharmacokinetic and pharmacodynamic profiles in preclinical settings [28–31]. Importantly, most of these drugs and drug candidates do not have any self-reporting ability and are therefore difficult to track noninvasively in real-time which makes their preclinical evaluation very challenging.

## Imaging modalities for real-time tracking of molecules in biological systems

Molecular imaging such as optical imaging (fluorescence and bioluminescence), magnetic resonance imaging (MRI), single-photon emission computed tomography (SPECT) and positron emission tomography (PET), to name a few, are powerful techniques for noninvasive visualization of physiological and pathophysiological processes in living systems at cellular and even molecular level [37–39]. Exploiting the specific reporting properties of a molecular probe for image contrast, these techniques enable real-time monitoring of biological processes, disease progression and characteristics, therapeutic efficacy of drugs etc. Each technique has its own benefits and limitations. Therefore, selection of appropriate imaging technique is purely based on the nature of the problem.

Optical or fluorescence imaging allows monitoring the behavior of a luminescent molecule in cells or in small animals with excellent spatial and temporal resolution [40]. The biological specimen is irradiated with a specific wavelength of light to excite the luminescent probe and the emitted light is then recorded. Its high sensitivity allows the visualization of even single molecules together with the possibility of monitoring rapidly changing biological events. Previously, the realm of optical imaging was limited to organic fluorophores, however, recent advancements are being driven towards the development of luminescent lanthanide and transition metal complexes [41]. The limited tissue penetration depth of light sometimes doesn't allow for optical imaging of probes in deep tissues or large subjects [42].

MRI is a highly versatile imaging technique. It is based on magnetic and radio frequency technology that excites and detects the change in the direction of the rotational axis of protons found in the water of living tissues [43]. This technique has high spatial resolution and can produce precise 3D positioning of diseased sites. However, for the detection to be more effective, certain contrasting agents such as Gd (III)-DTPA (DTPA = diethylenetriamine pentaacetate) need to be administered additionally to lower the detection time and to enhance the image contrast [44].

In SPECT imaging, a radioactive reporter probe (such as ^111^In and ^177^Lu complexes) is first injected into the patient and the emitted γ-ray photons from the tracer are recorded to identify the location of the probe [45]. This is a relatively affordable technique which is routinely used in the clinic as well as in translational research. The principle of PET is similar to that of SPECT except that γ-ray photons are not directly detected. Rather, the ejected γ-ray photons collide with electrons in the surrounding tissues leading to formation of two 511 keV photons which travel with a trajectory 180° apart and reach the circular detector. Importantly, since radioactive tracers do not require any light-mediated excitation, they are ideal for deep tissue tumor imaging.

Infrared (IR) imaging which uses the principle of vibrational spectroscopy is emerging as one of the non-destructive and powerful bioimaging techniques for tracking biologically relevant molecules in cells and tissues. It involves mid-IR (650–3300 cm^−1^) excitation of a particular bond or functional group of the target molecule in the vibrational levels [46,47]. Since most molecules possess vibrational modes lying within mid-IR, this technique can in principle be applied to investigate wide variety of analytes in biological systems [47,48]. It is a relatively safe technique compared to others as it does not require ionizing radiation or high energy photons or particles which may cause unwanted damage to cells or organelles. Moreover, it does not require electronic excitation, therefore photobleaching of the analyte does not occur [47]. Poor spatial resolution and signal-to-noise (S/N) ratio are critical drawbacks of mid-IR imaging using traditional thermal source. However, the technique has evolved and as demonstrated recently, the resolution as well as S/N ratio can be improved significantly by coupling a synchrotron radiation source to the IR microscope [46]. This synchrotron radiation FTIR spectromicroscopy (SR-FTIR SM) allowed imaging of living cells with a spatial resolution in the low micron range. In particular, this technique is extremely useful for tracking non-fluorescent metal-carbonyl based therapeutics that absorb strongly in between 1800 and 2100 cm^−1^ where no biomolecule absorbs [47].

It is evident that these molecular imaging modalities offer the possibility of repetitive, uniform, noninvasive real-time monitoring of a probe inside living subjects and therefore have the potential to play an important role in drug discovery and development process. Lack of knowledge on intracellular target, biodistribution and organ accumulation, systemic circulation time, pharmacokinetics, pharmacodynamics and excretion profile are main bottlenecks for clinical translation of new metal-based drug candidates [49]. Installation of an imaging modality within a metal-based drug will offer the possibility to track the same in real-time and facilitate the cellular, preclinical and clinical studies of the candidate.

## Theranostics comprising optical imaging

Optical imaging is the most widely used imaging modality due to the ease of its use, low cost and excellent biocompatibility. It employs radiation in visible or near-IR wavelength thereby alleviating the harmful effects caused by low wavelength radiation. One can access different colors and undertake multiplex analysis of different parameters simultaneously [50]. Metal-based optical theranostics are broadly of two types: (i) inherently luminescent therapeutically active metal complexes and (ii) therapeutically active metal complex attached to a luminescent probe (theranostic conjugates). In the later class, the metal complex acts as the therapeutic entity (cytotoxic agent) while the luminescent probe acts as the imaging entity (diagnostic entity). Typically, the luminescent probe is an organic fluorophore or another metal complex. The fluorophore should exhibit excellent optical properties (strong absorbance and relatively sharp fluorescence peaks with high quantum yield), biocompatibility and resistance to photobleaching. In the past few years, transition metal complexes have emerged as attractive alternatives to organic fluorophores for imaging purposes. These complexes have excellent photostability which allows for sustained and repetitive measurement over a long period of time. Their large Stokes’ shift ensures that the incident and emitted radiations are well separated [51,52]. Furthermore, the optical properties of these complexes could be tweaked by changing the ligand environment around the metal center. In this section, we will discuss a few prominent examples of metal-based optical theranostics with a particular emphasis on their design principle and biological studies in cells or in animals. For exhaustive examples of optical theranostics and theranostic conjugates including those of small-molecule metal-based optical theranostics, the readers are directed to consult some excellent reviews published recently [53–59]. For the sake of clarity, we have decided to classify the examples based on the metal center being employed.

### Cytotoxic inherently luminescent theranostics

#### Rhenium-based theranostics

The radioactive isotope of rhenium (Re), viz, ^186/188^Re has been used in the clinical treatment of cancer. A number of organometallic rhenium complexes have been employed as cell imaging agents and photocatalysts [60]. Re(I) tricarbonyl core [Re(CO)_3_] is frequently used in biological applications. These molecules can covalently bind to DNA nucleobases just like their Pt counterparts [61–63]. The ligand substitution kinetics of ‘(CO)_3_Re-Cl’ moiety is of the same order as that of cisplatin [64,65]. The [Re(CO)_3_] core is further endowed with rich spectroscopic properties. The triplet-based luminescent emission in the yellow region (560–590 nm) of these molecules has been used for cellular imaging [60]. Moreover, the distinct Re-CO stretching frequency enables their tracking in biological systems using IR spectroscopy (*vide infra*) [47].

**Figure 3:**
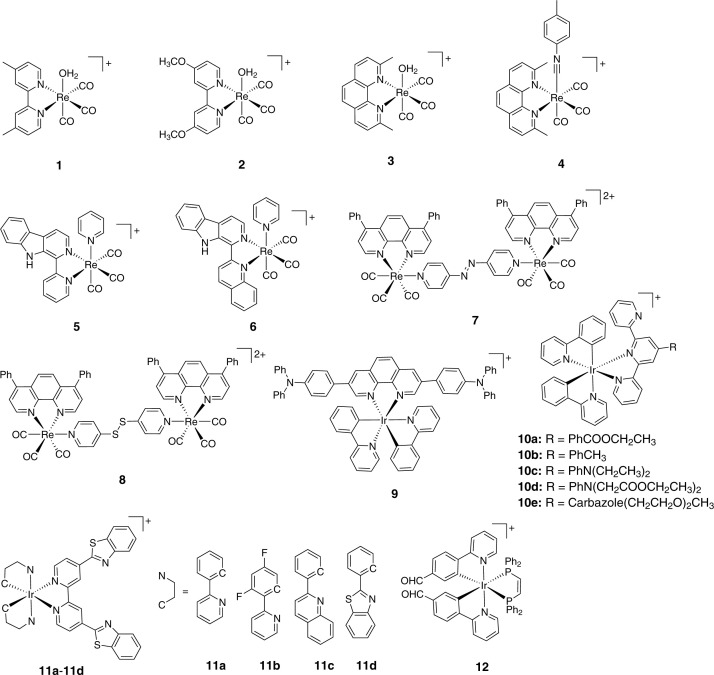
Cytotoxic, inherently luminescent Re(I) and Ir(III)-based theranostics

Wilson et al*.* reported a series of Re(I) complexes of the general formula *fac*-[Re(CO)_3_(N-N)(OH_2_)]^+^ where N-N is polypyridyl bidentate ligand (e.g. **1**-**3** in Figure 3) [65]. The complexes had different lipophilicities based on the nature of the bidentate ligand. *In vitro* anticancer activity of the compounds was evaluated by colorimetric 3-(4,5-dimethylthiazol-2-yl)-2,5-diphenyltetrazolium bromide (MTT) assay. Preliminary screening in HeLa cells indicated that **3** is the most cytotoxic compound with an IC_50_ value of 1.2 ± 0.2 µM and higher potency than cisplatin (IC_50_ = 3.0 ± 1.2 μM). Further, it was found that compound **3** could overcome cisplatin resistance in cancer cells. Importantly, the inherent luminescent property of this class of molecules allowed studying cellular uptake mechanism and intracellular organelle localization of **3**. Flow cytometry indicated active transport of **3** via endocytosis. Confocal microscopy suggested vacuole formation which were of endosomal-lysosomal origin. Additionally, although **3** was able to stall HeLa cells in their G2/M phase, no apparent increase in ROS or depolarization of mitochondrial membrane potential (MMP) was noticed. To gain further insight into the mechanism of cell death, the authors carried out cytotoxicity studies in HeLa cells in the presence of various cell death inhibitors. Results confirmed that **3** exerts its anticancer activity via a novel mechanism which cannot be characterized as apoptosis, necrosis, paraptosis or autophagy. The authors then went ahead and synthesized a radioactive technetium (^99m^Tc) analogue of **3** for *in vivo* biodistribution study in naïve C57B16 mice. This is an excellent feature of Re-based therapeutics, where ^99m^Tc analogue can be synthesized easily without perturbing the structure, charge, solubility etc. and can be used for *in vivo* pharmacokinetic evaluation in the early developmental stage (*vide infra*). Results suggested rapid renal and hepatic clearance for **3** and its ^99m^Tc analog. Metabolite analysis of **3** using HPLC-ICP-MS revealed the presence of intact **3** *in vivo* at all time points, indicating that the compound could reach tumor site prior to decomposition. Interestingly, the authors, in a follow-up paper reported that **3** is capable of inhibiting tumor growth in patient-derived ovarian cancer tumor xenografts upon injection via the tail vein at a dosage of 10, 20 and 30 mg/kg for a period of 31 days. Moreover, **3** preferentially accumulates in the mitochondria as indicated by ICP-MS analysis of cells treated with the compound [66]. This was contrary to the fluorescence microscopy based findings, which did not indicate any mitochondrial accumulation [65]. It was inferred that the surrounding pH affected the speciation of the complex, which, in turn, had implications on the photophysical properties of the Re(I) core.

The same group developed another rhenium(I) tricarbonyl complex containing a chelating polypyridyl ligand and an axial isonitrile ligand (**4**, Figure 3) as an anticancer candidate [67]. The complex was stable in solid and solution phase for weeks. Compound **4** had comparable or higher cytotoxicity as compared to cisplatin in a panel of cancerous cell lines. The mechanism of cell death induced by this complex was unraveled by determining its cytotoxicity in A2780 cell line in the presence of inhibitors of various established cell death pathways. The pan-caspase inhibitor ZVAD-FMK significantly decreased the cytotoxicity of **4**, indicating apoptosis as mechanism of cell death. Western blotting analysis of apoptosis marker proteins caspase 3 and cleaved PARP further corroborated the apoptotic cell death mechanism. The fluorescent property of **4** enabled tracking its localization in cells. Partial colocalization with the LysoTracker Red dye and GalT-dsRed fusion protein was observed. The compound also altered mitochondrial morphology which was ascribed to the mitochondrial fission process. Treatment with **4** resulted in a large increase in autophagosome marker LC3II expression relative to LC3I expression which pointed toward autophagy. Since mitochondrial fission, autophagy and ER stress are connected, the authors directed their investigations to examine ER stress in cells treated with **4**. Western blot studies indicated the phosphorylation of eukaryotic initiation factor 2α (eIF 2α). Puromycin incorporation assay in A2780 cells treated with **4** indicated lower rates of protein translation. Compound **4** also induced protein misfolding in HeLa cells as determined by thioflavin T (ThT) assay. Overall, it was inferred that the candidate exerts its antiproliferative activity by triggering unfolded protein response, ER stress, autophagy and apoptotic cell death. It is worth mentioning that **4** induces ER stress without ROS generation.

Mao et al*.* reported the synthesis and detailed biological investigations on a series of antiproliferative luminescent Re(I) tricarbonyl complexes with β-carboline ligands [68]. The complexes were shown to be stable in PBS/DMSO system at room temperature for at least 24 h. The intracellular localization of these complexes was examined by using confocal microscopy. In A459 cells, the confocal images showed that the phosphorescent signals of **5** and **6** had good overlap with LysoTracker Green but not with MitoTracker Green. This suggested that the complexes accumulated in the acidic lysosomes post cellular internalization. The *in vitro* cytotoxicity of these complexes was tested against several cancer cell lines. They exhibited marked activity against the cisplatin-resistant A549R cell line which signified their potential of circumventing cisplatin resistance. Compound **6** led to lysosomal dysfunction as was inferred using acridine orange (AO) staining and Magic Red cathepsin detection kit. These experiments pointed to the compromised lysosomal integrity and reduced cathepsin B activity. Furthermore, increased levels of LC3B-II were observed upon treatment of A549 cells with **6** which indicated induction of autophagy. Moreover, cells treated with 4 µM **6** showed positive Annexin V binding and PI staining. However, the cell viability of **6** treated cells was not altered in the presence of pan caspase inhibitor ZVAD-FMK, indicating caspase-independent apoptosis. The expression of reactive oxygen species (ROS) was then studied. Treatment of cells with **6** led to enhancement in fluorescence of DCF, indicative of increased ROS production. It was further corroborated by flow cytometry analysis. After analyzing the cell death mechanism under *in vitro* conditions, the authors proceeded to *in vivo* studies. Accordingly, **6** was injected intraperitonially after every 3 days at a dosage of 5mg/kg in nude mice bearing A549 tumor xenografts for a period of 21 days. The complex could inhibit tumor growth without any significant change in body weight of animals, indicating low systemic toxicity.

The same group has reported two binuclear rhenium (I) tricarbonyl complexes of the general formula [Re_2_(CO)_6_(dip)_2_L] (PF_6_)_2_ (dip = 4,7-diphenyl-1,10-phenanthroline; L = 4,4’-azopyridine (**7**) or 4,4’-dithiopyridine (**8**) [69]. The molecules were designed such that the liphophilic Re-moiety would facilitate cellular uptake and impart mitochondrial targeting while the azo or disulphide linkage would react with intracellular reductants. Compound **8** was shown to exhibit a better emission capability *in vivo* after intra-tumoral injection. Both complexes reacted rapidly with GSH and had a half-life less than 1 minute. The compounds showed good cytotoxicity *in vitro* when tested against a panel of cancer cell lines including A549R (IC_50_ values 8.3 ± 0.1 μM for **7**, 2.1 ± 0.1 μM for **8** and 51.3± 1.9 μM for cisplatin). Further, **7** and **8** could also inhibit colony formation *in vitro.* Confocal imaging and ICP-MS suggested that the compounds localized in mitochondria post cellular uptake. The complexes depolarized mitochondria, damaged mitochondrial DNA (mtDNA) and reduced oxygen consumption rate (OCR) which suggested an impaired mitochondrial bioenergetics status and cellular respiration. Subsequently, ROS production was studied and an increase in the superoxide (O_2_^.−^) level was also observed. Next, metabolomics analysis was employed on HeLa cells treated with **7** and **8** to analyze potential drug-related metabolites and pathways. The study indicated that the molecules could disturb GSH metabolism. The mechanism of cell death was further investigated by treating HeLa cells with the compounds in the presence of inhibitors of established cell death mechanisms. It was observed that the cell viability increased when the cells were pretreated with either necrostatin-1 (necroptosis inhibitor) or ZVAD-FMK (pan-caspase inhibitor). This suggested that the complexes induced apoptosis and necroptosis simultaneously. The authors then tested their molecules under *in vivo* conditions in a mouse xenograft model by injecting **7** and **8** intratumorally at a dosage of 5 mg/kg. Both **7** and **8** could inhibit tumor growth with a ratio of 44 ± 15 and 50 ± 16%, respectively, which is less effective than CDDP (30 ± 14%). Further, no pathological changes were observed in the organs from each group which suggested that the complexes do not produce severe side effects. It is worthy to note that despite extensive research being done in the design and development of rhenium-based anticancer molecules, only nine candidates were tested *in vivo* till date [70].

#### Iridium-based theranostics

Cyclometallated Iridium (III) (Ir) complexes have emerged as a promising class of theranostic agents due to their excellent antiproliferative activities and inherent luminescent properties. They exert novel modes of action such as catalytic interference of cellular redox balance and induction of non-apoptotic cell death thereby overcoming Pt chemoresistance [71]. They have favorable photophysical properties, e.g., high stability in physiological conditions, high emission quantum yields, large Stokes’ shift, resistance to photobleaching and long emission lifetime [60,72]. These phosphorescent Ir complexes show an instinct to localize in the mitochondria [73]. However, their intracellular localization can be tuned by varying the ligands around the metal center and other subcellular organelles like lysosome [74], lipid droplet [75] and endoplasmic reticulum [76] can be targeted.

Yang et al*.* reported a cationic Ir(III) complex with a donor-π-donor type ligand for application in photodynamic therapy (PDT) of cancer [77]. The viability of cells was greater than 85% at a concentration of 50 µM of **9** in the dark. The low toxicity could be due to either low uptake or an inherently noncytotoxic nature of the molecule. To understand this, the luminescent property of the molecule was utilized to visualize **9** in HeLa cells. Confocal microscopy suggested that the complex was efficiently taken up by the cells within 2 h and localized exclusively in the cytoplasm, confirming the true nontoxic nature of the compound in the dark. This is one of the most desirable properties for an ideal photosensitizer (PS). The molecule could efficiently generate ^1^O_2_ upon irradiation with an NIR laser (730 nm). Encouraged by this observation, the authors tested the photodynamic therapy (PDT) capability of the molecule under *in vivo* conditions. HeLa tumor bearing mice were intratumorally injected with the complex (30 mg/kg body weight) and then irradiated by a 730 nm CW laser (0.2 W/cm^2^) for 10 min every 2 days. The control groups of mice were treated with either the same dose of **9** (in dark) or irradiated with 730 nm CW laser alone without **9**. TUNEL staining and HO-1 staining of the tumor tissues after 14 days pointed to apoptotic mode of tumor cell death. Unfortunately, the authors did not observe any appreciable PDT effect *in vivo*, which might be due to small nonlinear absorption coefficient of **9**.

Tian et al. developed a panel of terpyridine-based Ir(III) complexes for 2-photon PDT applications (**10a**-**10e**, Figure 3) [78]. It was found that **10a** was not cytotoxic under dark conditions towards HepG2 cells as indicated by ∼85% viability after treatment with 15 μM for 24 h. Upon UV irradiation a significant decrease in cell viability was observed (∼4% viability). The luminescent properties of these molecules offer the opportunity to study how the terminal substituents modulate the biological properties of this class of molecules using confocal microscopy. Colocalization experiments with organelle tracker indicated that while **10a** localized in nucleus, the other derivatives **10b**-**10e** localized in mitochondria. Interestingly, **10a** and **10b** share the same backbone structure but have slightly different terminal substituents on the terpyridine ligand. This variation of terminal substituents also had implications on their cellular uptake mechanism. While **10a** triggered microtubule-dependent endocytosis, **10b** entered via clathrin-mediated endocytosis. The PDT efficiency of the complexes was then evaluated on HepG2 cells. Interestingly, **10a** could migrate from nucleus to the mitochondria and continue to induce dual-damage under light exposure. To further assess the capability of **10a** as a potential PDT agent, *in vivo* mouse experiments were carried out. The compound could significantly inhibit the growth of solid tumor and importantly the efficacy was comparable to that of the commercial PDT agent Ce6. However, *in vivo* imaging experiments were not performed to demonstrate the diagnostic property of this molecule.

Chao et al*.* have designed complexes **11a**-**11d** (Figure 3) using benzothiazole substituted ligands [79]. The complexes were stable in fetal bovine serum (FBS) and cell culture media up to 48 h and exerted potent antiproliferative activities with sub-nanomolar IC_50_ values against various cancer cell lines including Pt and mitoxantrone, doxorubicin-resistant cancer cells. Confocal microscopy was performed to understand the intracellular localization of the complexes. The study revealed that the complexes localized in the mitochondria. This was further corroborated using ICP-MS analysis of Ir content in isolated mitochondria from compound treated cells. It was also determined that the compounds were internalized by endocytosis process. ChChd3 is an abundant protein residing in the mitochondrial inner membrane. This protein is essential for maintaining mitochondrial cristae structure. In A549R cells treated with a selected candidate **11a**, the ChChd3 content decreased significantly in a dose-dependent manner which reflected an injury to the mitochondrial inner membrane [80]. Complex **11a** caused dissipation of MMP and inhibited ATP production due to generation of reactive oxygen species (ROS). Further, Western blotting experiment revealed that the ratio of proteins bcl-2 to bax increased. This trend is not characteristic of regular apoptosis but is consistent with another form of programmed cell death known as oncosis [81–83]. Surprisingly, the content of cytochrome C in the cytosol did not increase despite the severe loss of MMP. The whole-cell caspase 3/7 expression level did not increase. Also, coincubation of the compounds with caspase inhibitors Ac-DEVD-CHO and Z-VAD-FMK did not lead to any significant change in cell viability. Detailed morphological analysis of the cells by using confocal laser scanning microscopy (CLSM) and TEM coupled with viability assays in the presence of inhibitors of established cell death mechanisms ruled out apoptosis, paraptosis, necrosis, necroptosis and autophagy as possible modes of cell death. CLSM imaging further demonstrated that the complexes could lead to collapse and breakdown of the cytoskeleton in a dose-dependent manner. Subsequent Western blotting analysis revealed the increase in expression of two proteins calpain 1 and porimin which are hallmarks of oncotic cell death.

Mao et al*.* synthesized a series of six phosphorescent cyclometalated Ir(III) complexes with the general formula [Ir(C–N)_2_(P–P)]PF_6_, where C–N = 2-phenylpyridine or 4-(2-pyridyl)benzaldehyde and P–P = bis-(diphenylphosphino)methane or bis- (diphenylphosphino)ethane or bis-(diphenylphosphino)ethylene [84]. The design principle was based on the introduction of rotationally flexible groups into the emissive iridium complexes. The complexes possessed viscosity-sensitive two-photon absorption (TPA) properties. So, it was expected that these complexes would report the changes in the subcellular microenvironment while exerting their anticancer activity. Among all compounds tested **12** (Figure 3) showed the best liner response to viscosity as well as excellent antiproliferative activities in A549, HeLa and LO2 cells. The intrinsic emission properties were utilized to study cellular accumulation and organelle localization of **12**, which shed light on anticancer mechanism of action of this class of compounds. Confocal microscopy and ICP-MS analysis suggested that **12** localized solely in the mitochondria. As expected, **12** dissipated mitochondrial membrane potential (MMP), decreased ATP production and basal respiration level in A549 cells. Subsequent evaluation of the ROS levels using the H_2_-DCFDA dye indicated a massive upregulation of ROS. Since **12** exhibited a linear response between its lifetime and environmental viscosity, therefore, two-photon phosphorescence lifetime imaging microscopy (TPPLIM) was employed to get a real-time data on the mitochondrial viscosity. Importantly, the authors have investigated the two-photon imaging capability of **12** *in vivo* using a zebrafish model. The compound can be visualized easily in different body parts of the animal and phosphorescence lifetimes revealed varied viscosities in different tissues of the zebrafish larvae. Cumulatively, these results suggest that **12** has the potential to be an excellent theranostic agent.

#### Ruthenium-based theranostics

Ruthenium-based anticancer agents have a lot of interesting properties like good selectivity towards cancer cells, suitable ligand exchange kinetics and variable oxidation states which are accessible under biological conditions. As discussed above, four ruthenium complexes **NAMI-A, KP1019**, **NKP-1339** and **TLD1433** have entered various phases of clinical trials and many candidates are in the early developmental stage [85–87]. However, in the context of theranostics, Ru (II) polypyridyl complexes are particularly attractive. They possess metal-to-ligand charge transfer red emission, large Stokes’ shift, long emission lifetimes and two/multiphoton absorption properties [88]. Selectivity of the complexes towards cancer cells or subcellular organelles could be brought about by either ligand modification or conjugation with certain targeting vectors. Here, we’ll discuss a few examples of Ru based theranostics with particular emphasis on their anticancer activity and elucidation of their biological mechanisms of action using optical imaging.

**Figure 4:**
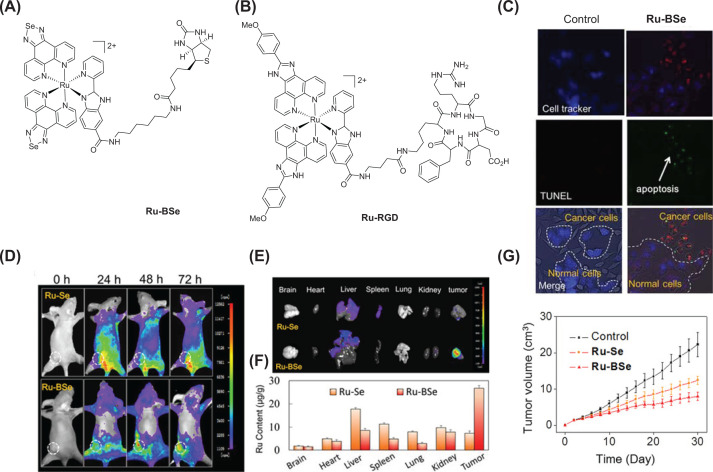
Ru(II)-based targeted theranostics (**A** and **B**) Structures of selected examples of Ru-based targeted theranostics **Ru-BSe** and **Ru-RGD**. (**C**) Fluorescent microscopy revealed cancer cell selective uptake and induction of apoptosis by the treatment of **Ru-BSe** (20 μm) for 24 h in HeLa (cancer cells) and L02 (noncancerous cells) co-culture model was examined by TUNEL-Cell tracker blue co-staining. (**D**) Precise tumor diagnosis using **Ru-BSe** conjugate *in vivo*. Targeted **Ru-BSe** (4 μmol kg^−1^) was administered in HeLa xenografts bearing nude mice and fluorescence imaging at different time points were performed to monitor the accumulation and distribution of the theranostic. Nonbiotinylated Ru(II) complex **Ru-Se** was included as negative control. Fluorescent filter sets (excitation/emission, 500/650 nm) are used for *in vivo* fluorescent imaging. (**E**) Fluorescent images of ex vivo-dissected organs (brain, heart, liver, spleen, lung, kidney, and tumor tissue) of the **Ru-Se** and **Ru-BSe**-injected xenograft mice after 72 h tail-vein injection. (**F**) Biodistribution of Ru in main organs after 30-day treatment of Ru(II) complexes in HeLa xenografts nude mice by using ICP-AES. (**G**) *In vivo* anticancer efficacy of **Ru-BSe** and **Ru-Se** in in HeLa xenograft mice model (dose: 4 μmol kg^−1^ every 2 days). Reprinted with permission from the reference [89]. Copyright 2018, Wiley.

Chen et al*.* synthesized a Se-containing Ru conjugate (**Ru-BSe**, Figure 4A) covalently linked with a cancer targeting molecule, namely, biotin [89]. Phosphorescent emission property of this conjugate allowed for real-time tracking and imaging of the molecule inside cells as well as in mice xenograft model. The complex was stable in DMSO and human plasma for a period of 72 h. The cytotoxicity assays revealed that **Ru-BSe** had a IC_50_ value comparable to that of cisplatin in a panel of cancer cell lines. Next, confocal microscopy was employed to determine the intracellular localization of the complex. The complex accumulated in the cytoplasm after an incubation time of 6 h with a good colocalization with the mitochondria (Pearson correlation coefficient = 0.90).

The compound caused dose-dependent disruption of the MMP in HeLa cells. Western blotting assay revealed the downregulation of Bcl-2 (anti-apoptotic proteins) and an upregulation of Bax (pro-apoptotic proteins). The ROS levels in cells treated with **Ru-BSe** increased within 4 h. This increased ROS level led to ER stress as evidenced by increasing level of ER stress related proteins p-PERK and CHOP. Induction of apoptotic cell death mechanism was confirmed by flow-cytometric analysis and analyzing the levels of activated caspase-3, caspase-9. The authors then went ahead and tested the cancer targetability of the conjugate *in vitro* as well as *in vivo*. A co-culture of HeLa (biotin receptor positive cancer cells) and L02 (biotin receptor negative noncancerous cells) cells was treated with **Ru-BSe** and fluorescent property of the molecule was exploited to visualize the molecule inside cells. Results suggested **Ru-BSe** had the ability to selectively accumulate in and annihilate cancer cells without affecting the normal cells (Figure 4C). Then **Ru-BSe** was administered through tail-vein at 4 µmol/kg dose in mice bearing HeLa-xenograft and fluorescence imaging was performed at different time points to monitor tumor accumulation and biodistribution *in vivo*. The tumor accumulation of **Ru-BSe** reached a maximum after 72 h with very low accumulation in other tissues, allowing precise diagnosis of tumor in mice (Figure 4D). The *ex vivo* fluorescence imaging and ICP-AES of excised tumor and organs further confirmed selective tumor accumulation of **Ru-BSe** over other organs (Figure 4E,F). As shown in Figure 4G, **Ru-BSe** showed excellent *in vivo* antitumor efficacy with a 64% tumor growth inhibition at day 30. Further, the molecule did not cause any adverse side effects over the course of treatment. The tumor accumulation and efficacy of the nonbiotinylated analogue **Ru-Se** were significantly lower as compared to those of **Ru-BSe**, highlighting the importance of targeting vectors for success of theranostic drugs. This study demonstrated the design and successful evaluation of a targeted metal-based theranostic construct.

Zhao et al*.* reported a targeted polypyridyl Ru prodrug for cancer therapy [90]. The molecule employed a cyclic Arg-Gly-Asp (RGD) peptide as the targeting vector (**Ru-RGD**, Figure 4B). The cytotoxicity of the molecule was evaluated in a panel of cervical cancer (CaSki, SiHa and HeLa) and normal (Ect1/E6E7) cell lines. IC_50_ value of **Ru-RGD** in CaSki cells was 3.8 μM, which is comparable with that of the clinical metallodrug cisplatin (4.2 μM). A co-culture of CaSki and Ect1/E67 cells was assessed using TUNEL staining assay to verify selective induction of apoptosis by **Ru-RGD** to cancer cells. Furthermore, flow cytometry data confirmed that the percentage of apoptotic CaSki cells was 24.6% compared with 5.8% for Ect1/E6E7 cells. That **Ru-RGD** selectively accumulates in the cancer cells could also be visualized using confocal microscopy. The two-photon-induced luminescence of the theranostic **Ru-RGD** was examined in 3D CaSki multicellular tumor spheroids. It was found that the two-photon luminescence of **Ru-RGD** was more suited to deep tissue imaging. Further, **Ru-RGD** could also suppress the spheroid growth with better efficiency compared with cisplatin. Encouraged by these results the authors carried the molecule ahead for *in vivo* studies. The time-dependent biodistribution of the molecule was carried out in nude HeLa xenograft mice at a dosage of 4 μmol/kg of the complex. After 36 h majority of the injected complex could be observed in the tumor demonstrating excellent targetability of the theranostic. Finally, the complex was shown to inhibit tumor growth with an inhibition rate of 74.3%. No death or obvious changes in body weight of the mice were observed after administration of the prodrug.

### Miscellaneous metal-based theranostic conjugates

So far, we have discussed about metal-based theranostics whose intrinsic luminescent properties were utilized to investigate the cytotoxic mechanism of action *in vitro*. In this section, we will briefly discuss about theranostic conjugates where a non-fluorescent cytotoxic metal center has been conjugated to a luminescent metal complex or an organic fluorophore to facilitate imaging. We have chosen a few selected examples to illustrate the design principle of such conjugates.

**Figure 5:**
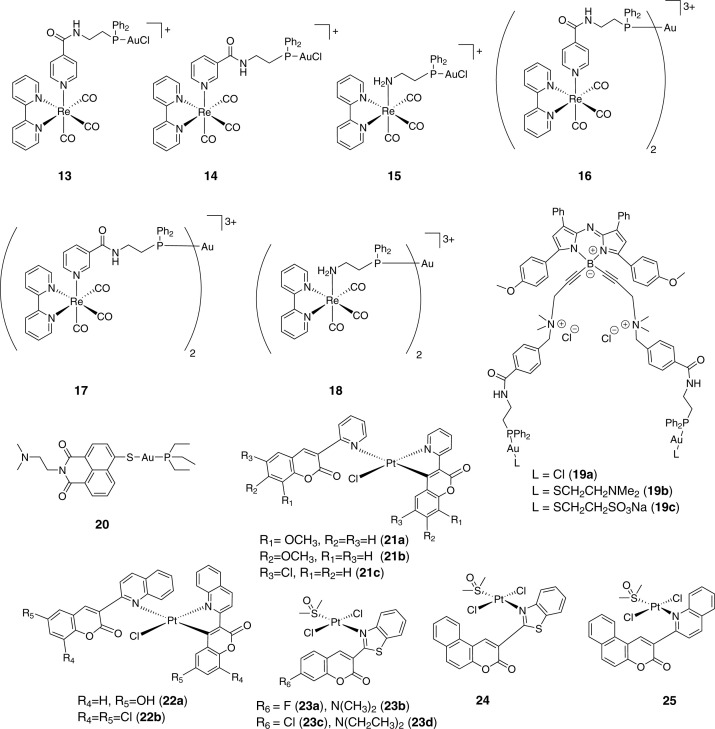
Selected examples of miscellaneous metal-based theranostic conjugates

Gimeno et al*.* synthesized a series of heterobimetallic and heterotrimetallic complexes incorporating a luminescent Re(I) species and a bioactive Au(I) derivative. A ditopic P,N-donor ligand (L) was used as a linker between both metals, affording six new bipyridine (bipy) Re(I)/Au(I) hetero-metallic complexes **13**-**18** (Figure 5) [91]. After ascertaining the stability of the complexes in PBS, the authors went ahead with the preliminary cytotoxicity studies. The complexes were shown to have moderate cytotoxicity in A549 cell line with IC_50_ in the range of 35 to 75 µM. The Re core mainly functions in lending emissive properties to the molecule, as observed for other heterobimetallic Re(I)/Au(I) complexes [92]. The heterotrimetallic compounds (**16**, **17**, **18**) exhibited higher cytotoxicity, with their IC_50_ values being almost half of their bimetallic counterparts. Next, oxidative stress imparted to the cells by the molecules was quantified using H_2_-DCFDA assay. Flow cytometry analysis revealed higher 2′,7′-dichlorofluorescein (DCF) fluorescence in compound treated cells, corroborating an increase in the level of intracellular ROS. Then luminescent properties of the Re(I) core were utilized to understand the intracellular localization of these complexes. A549 cells were treated with the complexes (25 µM) together with a commercial nuclear dye DRAQ5 (2 µM) and imaged using fluorescence microscopy. Complexes **16** and **17** could be visualized, however the rest could not be visualized. The lack of visualization of the complexes was ascribed to the quenching phenomenon in biological medium. Complexes **16** and **17** showed similar behavior in terms of their intracellular localization, with clear accumulation near the edge of the inner cell membrane and in areas within the nucleus. The compounds were internalized in the cell by a passive diffusion process. The compounds were shown to interact with DNA, however, their precise mechanism of action was not clear. Further examples of heterobimetallic theranostics of this class have been reported by the Casini [93], Patra [94], Gornitzka [95] and also reviewed recently [96,97].

Bodio et al*.* recently reported three near-infrared (NIR) optical theranostic systems comprising of Au(I) as therapeutic unit and aza-BODIPY as imaging unit (**19a**-**19c**, Figure 5) [98]. In contrast to the above discussed example, these molecules contain an organic fluorophore. Aza-BODIPY fluorophores are chemically and photochemically stable, exhibit good quantum yield and, unlike many fluorophores emitting in NIR, are easy to functionalize. Low water-solubility is their key drawback.

The same group recently showed that the substitution of the fluorine atoms present on the boron atom of the aza-BODIPY core by ammonium arms could significantly improve the water-solubility and limit aggregate formation [99–101]. Preliminary cytotoxicity studies in 4T1, MDA-MB-231, CT26, SW480 cancer cell lines revealed that all complexes exhibited good antiproliferative activities with IC_50_ < 10 µM, which is rare for metal complexes coupled to a fluorophore. The quantification of cellular uptake of the molecules was performed by employing ICP-MS to determine gold content in compound treated cells. When the cells were treated with 5 µM of the complexes for 4 h, the uptake followed the order: **19a** < **19b** < **19c**. Thioredoxin reductase (TrxR) is a potential target of Au(I) anticancer agents. Among the three molecules, **19a** showed maximum inhibition of TrxR. Afterward, the optical properties of the theranostics were leveraged to understand their intracellular distribution. Confocal imaging was performed on human MDA-MB-231 cells and mouse 4T1 cells. The complexes mainly localized inside small cytoplasmic vesicles and close to the nucleus, at longer time they seemed to accumulate in Golgi apparatus. The molecules did not have any proclivity of localizing in the nucleus or mitochondria. The authors studied the anticancer efficacy, biodistribution and tumor diagnostic ability of **19a** *in vivo*. BALB/c mice bearing syngenic colon CT26 flank tumors were injected intravenously with a dose of 0.5 mg/kg body weight. The compound could inhibit tumor growth without any observable side effects. Furthermore, the real-time fluorescence tracking (λ_ex_ 685 nm and λ_em_ at 705 nm) suggested that the compound accumulated around the tumor site with a maximum around 4 h post injection. After 24 h of administration, most of the compound was found in the excretory organs such as liver, kidney and spleen.

A similar Au(I)-based theranostic containing a naphthalimide fluorophore moiety was reported by Ott et al*.* (**20**, Figure 5) [102]. Compound **20** had IC_50_ values of 2.6 ± 0.4 µM in HT29 and 1.3 ± 0.7 µM in MCF7 cells, which are comparable to those obtained for the standard drug auranofin. Fluorescence microscopy studies were done on HT-29 and MCF-7 cells exposed to **20**. The luminescence appeared to be located in specific areas of cytoplasm along with the nucleus. Atomic absorption spectroscopy (AAS) further revealed the accumulation of **20** in the nucleus of cells most likely due to the DNA targeting properties of naphthalimide ligand. The mechanism of action studies suggested TrxR as one of the potential targets. Further, a dose-dependent loss of mitochondrial membrane potential was observed in cells treated with **20.** Since TrxR is known to stimulate angiogenesis [103,104]. **20** was tested for its anti-angiogenic property in a zebrafish embryo model. At a maximum tolerated dose (MTD) of 0.1 µM, compound **20** could induce significant anti-angiogenic effects.

Qin and co-workers reported a series of eleven organo-Pt (II) complexes bearing quinoline-coumarin derivatives (**21**-**25**, Figure 5) [105]. The compounds were screened in A549, cisplatin-resistant A549/DDP, SK-OV-3, cisplatin-resistant SK-OV-3/DDP, HeLa, NCI-H460a cancer cell lines and in HL-7702 noncancerous cell line. All complexes displayed significantly higher antiproliferative activity compared with cisplatin and in some cases the IC_50_ reached nanomolar value (e.g. IC_50_ of **22a =** 0.10 ± 0.05 µM in A549/DDP cells). The luminescent property of the molecule was exploited to study intracellular accumulation of **22a** using confocal microscopy. Colocalization studies with DAPI and MitoTracker suggested preferential accumulation of the compounds in mitochondria. Western blot analysis confirmed upregulation of caspase-9, caspase-3, apaf-1 and cytochrome *c* and downregulation of bcl-2, indicating a mitochondria-mediated cell death in A549/DDP cell line. The authors then went ahead and carried out *in vivo* studies in HeLa tumor xenograft model. The molecule **22a** was injected at a dose of 2.0 mg/kg in every 2 days for 21 days. A decrease in tumor volume as compared with the control group was observed. Further, treatment with the molecule did not lead to any noticeable side effects in the mice. However, the imaging capability of the molecule was not tested *in vivo*.

## MRI-guided theranostics

**Figure 6:**
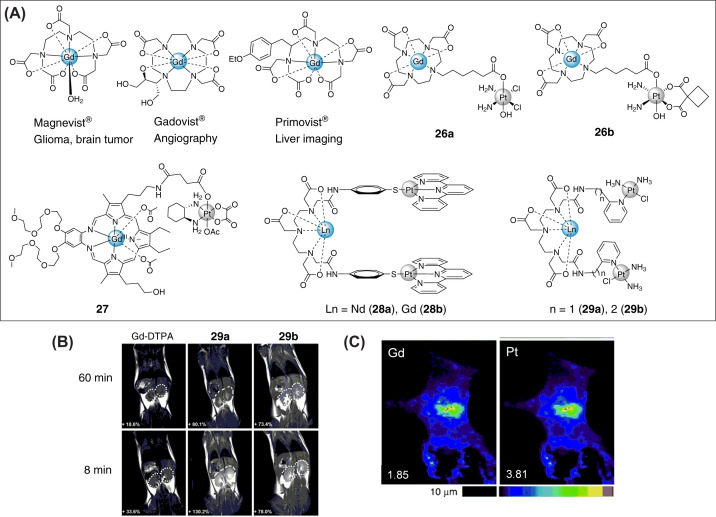
MRI-based bimetallic theranostic agents (**A**) Selected examples of a few Gd-based clinically used MRI contrast agents (Magnevist, Gadovist and Primovist) and Gd-Pt heterometallic MRI-based theranostics. (**B**) *In vivo T*_1_-weighted MR images of B6 mice at 8 and 60 min after the injection of **29a**, **29b** and Gd–DTPA at a dose of 0.1 mmol Gd / kg of body weight and the corresponding signal-decay curves. The circles indicate the area of the kidneys. Reprinted with permission from reference [129]. Copyright 2014, Wiley. (**C**) XRF elemental distribution maps for Gd, and Pt of a single A549 human lung carcinoma cell. Cells were treated with 5 µM of **28b** for 24 h. Maximal area densities are given in µgcm^−2^ for each element at the bottom of each map. Reprinted with permission from reference [128]. Copyright 2010, Wiley.

Magnetic resonance imaging (MRI) is one of the commonly used clinical diagnostic tools which produces multiplanar and true 3D cross section images of the region of interest in a patient’s body [106–108]. The technique primarily relies on the water present inside the body, in and around the tissues and organs to produce a contrasted image of a cross-section of that particular organ/tissue [109]. Aberrations like lesions or tumors can be easily identified/distinguished from a healthy organ/tissue. Sometimes the resolution of the image may not be sufficient, especially for small tumors or tissue lesions, then contrast agents are necessary. Contrast agents increase the resolution by enhancing image contrast [110]. They function by accumulating in a specific target site and shortening the relaxation times (of *β* to *α* transition) T1 and T2 of the excited nuclei [111] in that specific area thereby brightening up the target site and increasing the resolution. Paramagnetic metal complexes of gadolinium (^64^Gd^3+^, magnetic moment = 7.94 B.M.) and iron oxide (^26^Fe^3+^, magnetic moment = 5.92) are oftenly used for this purpose [112–121]. A few widely used Gd-based MRI contrast agents are Magnevist (for diagnosis of glioma and brain tumors), Gadovist (for Angiography) and Primovist (Liver diagnosis) are presented in Figure 6A [44,122,123]. Importantly, low toxicity, rapid renal clearance, and high stability make these agents ideal for imaging applications.

Typically, this class of theranostics are designed through covalent conjugation of Gd-based MRI contrast agents to metal-based anticancer agents [124]. Reports on such bimetallic MRI-based theranostics are relatively fewer in number and mostly limited to *in vitro* proof-of-concept experiments. We will discuss a few recent and key examples that establish the platform for future development of MRI-based bimetallic theranostics. The simplest example, in terms of design, of this class is that of Gd(III)-Pt(IV) conjugates **26a** and **26b** reported recently by Meade et al*.* (Figure 6A) [125]. The theranostics were constructed by coupling a Gd-DOTA (DOTA = 1,4,7,10-tetraazacyclododecane-N,N',N”,N“'-tetraacetic acid) MRI contrast unit to Pt(IV) chemotherapeutic prodrugs derived from either cisplatin or carboplatin. A C6 linker was used to connect the two moieties. Compound **26a** was found to have better antiproliferative activities as compared to **26b** in A2780, HeLa and MCF-7 cancer cell lines. As expected for Pt (IV) prodrugs, both compounds are less cytotoxic as compared to their respective Pt(II) drugs cisplatin and carboplatin. ICP-MS measurement of intracellular metal content of cells treated with compounds revealed that accumulation of Gd was 4-20 times higher than that of Pt. This was explained considering rapid intracellular reduction of Pt(IV) center and subsequent separation into individual Pt (II) and Gd (III) moieties which possessed differential efflux rates. Then the authors performed imaging experiments using cell-pellet as an approximate model of a 3D tumor to test the abilities of **26a** and **26b** to enhance the MRI contrast. Accordingly, A2780 and HeLa cells were incubated with **26a** and **26b** at a concentration equivalent to their IC_50_ values for 6 hours. MRI experiments at 7T magnetic field revealed that **26a** performed better than **26b** in enhancing the intracellular relaxivity, *R*_1_ (1/*T*_1_) which is directly proportional to contrast. **26a** increased the *R*_1_ by 36% (in A2780 cell-pellet) and 48% (in HeLa cell-pellet) compared with untreated control-cell pellets, demonstrating the theranostic ability of this bimetallic compound. However, the true theranostic potential of this class of compounds is yet to be demonstrated using *in vivo* xenograft models.

As discussed before, the discrepancy in accumulation of imaging unit and therapeutic agent can be addressed by reducing the separation kinetics using a relatively stable chemical linkage. In a recent report, Sessler and co-workers reported a series of Gd(III) texaphyrin-Pt complexes. The design was similar to that of Meade et al [125]*.* However, oxaliplatin-based Pt(IV) derivatives were used instead of cisplatin or carboplatin [126]. This modification significantly increased the stability of the bimetallic conjugates. For example, the best compound **27** (Figure 6A) was inert toward hydrolysis under physiological conditions. Moreover, stability experiments in reducing conditions (using glutathione) also reconfirmed excellent stability of **27**. The antiproliferative potency of **27** in A549 cells was shown to be 7-fold higher than the combination of individual Gd(III) and Pt(IV) units. The authors attributed the enhanced cytotoxicity to two parameters: (a) A more facile intramolecular electron transfer between the texaphyrin core and the Pt (IV) and (b) Enforced proximity (essentially a colocalization effect under the conditions of *in vitro* testing). It was also found out that **27** could induce apoptosis in both a p53-dependent and p53-independent manner. Also, **27** was able to induce/reactivate p53 function in 2780CP/Cl-16 Pt-resistant ovarian cell lines. Importantly, **27** exerted excellent i*n vivo* activity in both Pt-sensitive A549 xenografts as well as Pt-resistant patient-derived 0253 xenografts. Despite exhibiting excellent therapeutic potential *in vivo*, the diagnostic potential of **27** is yet to be tested using MRI.

Glover and co-workers published a hairpin-shaped luminescent neodymium (Nd)-Pt heterometallic complex **28a** (Figure 6A) [127]. It selectively accumulates inside the nucleus and the flat terpyridyl ends of the hairpin intercalate with nuclear DNA as confirmed by linear dichroism (LD) measurements. DNA intercalator metal complexes have been shown to exert anticancer properties [85]. Metallo-hairpin class of compounds of this type could be potent theranostic agents having therapeutic ends on the hairpin and an imaging unit in the central hairpin bend. Crossley and co-workers replaced Nd with Gd to obtain an analogous compound **28b** (Figure 6A) [128]. Similar to its Nd analog, Gd complex **28b** shows high accumulation in the nucleus of A549 cells. Importantly, **28b** reaches nucleus in its intact form and binds to DNA as visualized by synchrotron XRF elemental distribution maps of Gd and Pt (Figure 6C). Despite their stability in biological systems, there is not enough experimental data to confirm their anticancer potential. Additionally, the diagnostic potential of **28b** was not investigated using MRI.

Guo et al*.* replaced the Pt-terpyridyl moieties in **28b** with pyriplatin units to construct Gd-Pt_2_ metallohairpin anticancer theranostics **29a** and **29b** (Figure 6A) [129]. ICP-MS quantification of Gd and Pt in HeLa cells treated with **29a** and **29b** suggested that the compounds were capable of entering the cells. The complexes underwent partial dissociation inside cells within 24 h. The released Pt unit travelled to the nucleus and platinated DNA, whereas the Gd unit mainly remained in the cytoplasm. Both complexes exerted significantly lower cytotoxicity compared to cisplatin in MCF-7, A549 and HeLa cells. However, at higher concentration of the compounds (0.1 mM), similar efficacy to that of cisplatin was observed. It is important to note that this concentration is sufficient to produce an effective contrast effect for Gd-based contrast agents. Due to decreased rotational mobilities, **29a** and **29b** had significantly higher relaxivity compared with the standard clinically used MRI contrast agent Gd-DTPA. The diagnostic potential of the complexes was first demonstrated by acquiring images of **29a** and **29b** treated HeLa cell suspensions. Next, the imaging capability of the complexes was tested under *in vivo* conditions. Accordingly, B6 mice were injected with the complexes at a dose of 0.1 mmol Gd kg^−1^ of body weight. MRI of the animals 8 min post injection suggested that the heterometallic derivatives **29a** and **29b** showed more intense signal in the kidney (Figure 6B). Importantly, the contrast is significantly higher as compared to the control Gd-DTPA and the high level of intensity was maintained for more than 1 h. This is important in terms of renal imaging over extended periods of time. Since Pt drugs are known to induce nephrotoxicity, these compounds could be employed for noninvasive diagnosis of acute renal injury. Similar to attachment of chemotherapeutics, photosensitizers (PS) such as porphyrins and metal-porphyrins were conjugated with Gd(III) based contrast agents for MRI guided photodynamic therapy. Advances in the development of PS-Gd conjugates were recently summarized by Sour et al [130]*.*

## Theranostics comprising radioimaging

Radioactivity is used in nuclear medicine for both diagnosis and radiotherapy. The choice of modality is dependent on the radionuclide being employed. Diagnostic studies are carried out by using γ-ray emitters or positron emitters. On the other hand, therapeutic outcome is achieved by employing radionuclides emitting low-range highly ionizing radiation like α- or β-particles. Radionuclides which qualify to be termed as ‘theranostic radionuclides’ therefore, have a combination of both entities in a single molecule. These molecules could be further categorized into two broad classes depending upon the identity and mode of action of the cytotoxic entity. One could have a radioactive nuclide-radioactive nuclide theranostic combination wherein one isotope is the reporter entity and other is the therapeutic entity which exerts its cytotoxic activity by means of high-energy radiation. The other combination is a radioactive nuclide-cytotoxic entity construct. Here the cytotoxic entity obliterates the cancer cells via a ‘non radiative damage’ pathway. In this review we'll be restricting ourselves to the latter category, viz, molecules containing a cytotoxic metal center with a radioactive nuclide as the imaging entity and discuss a few prominent candidates of this class.

Typically, the radionuclide-based theranostics are tracked by employing either positron emission tomography (PET) or single photon emission computed tomography (SPECT). PET is usually preferred to SPECT for its superior spatial resolution and its possibility of quantifying several parameters.

**Figure 7:**
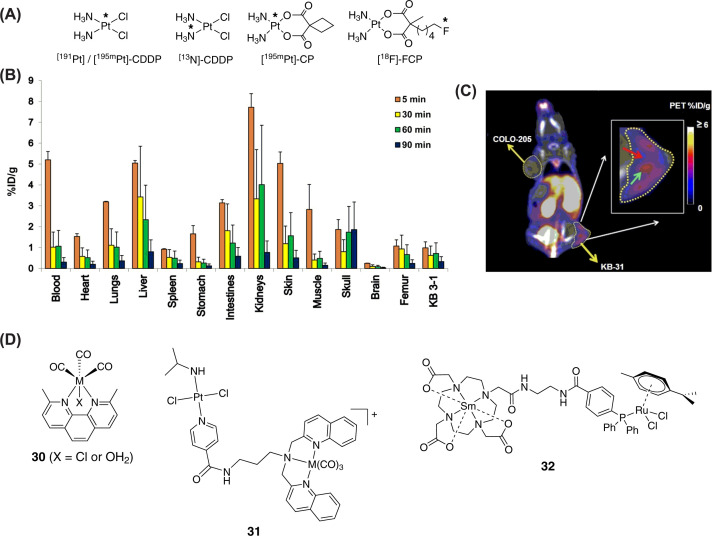
Radionuclide-based theranostics (**A**) Chemical structures of radioactive versions of cisplatin ([^191/195m^Pt]-CDDP and [^13^N]-CDDP) and ^18^F-carboplatin analog [^18^F]-FCP. (**B**) Biodistribution of [^18^F]-FCP in KB-3-1 cervical tumor xenograft–bearing adult female nude mice (*n* = 3 / time point) at 5, 30, 60 and 90 min after intravenous injection. Radiotracer uptake in %ID/g was determined by γ-counting. (**C**) [^18^F]-FCP (5.5–6 MBq) was administered through tail vein injection into the mice bearing KB-3-1 and COLO-205 xenografts. Imaging was performed 90 min after injection using a PET/CT scanner, and static image was reconstructed and analyzed. Images showed obvious differences in uptake of [^18^F]-FCP between KB-3-1 (2.6% ID/g) and COLO-205 (0.8% ID/g) tumors. Furthermore, heterogeneity of uptake was evident in KB-3-1 tumor (inset), showing regions of high (cyan arrow) and low (red arrow) [^18^F]-FCP uptake. Panels B and C were reproduced with permission from an original research article published in *J. Nucl. Med.* 2017, 58, 1997-2003. (**D**) Structures of Re/^99m^Tc congener pairs (**30**-**31**, M = Re or ^99m^Tc) and bimetallic PET/SPECT theranostic conjugates (**32** and **33**).

The simplest examples of such theranostics are the radioactive forms of anticancer drugs cisplatin and carboplatin such as [^191^Pt]-CDDP, [^195m^Pt]-CDDP, [^13^N]-CDDP and [^195m^Pt]-CP (Figure 7A) [131,132]. Since incorporation of radioactive Pt does not alter chemical structure and physicochemical properties, these radiolabeled drugs are true reporters of *in vivo* pharmacokinetics for the nonradioactive Pt drugs. Among the four radioactive isotopes of Pt (^191^Pt, ^193m^Pt, ^195m^Pt, ^197^Pt), ^195m^Pt is the most preferred one for preparation of radioactive Pt drugs due to its ideal γ energy spectrum (similar to ^201^Tl) which is suitable for imaging, ease of production from ^194^Pt and longer half-life (4 days) that is appropriate for efficient synthesis, quality control and *in vivo* radioimaging. Using [^195m^Pt]-CDDP and [^195mPt^]-CP, Wolf et al*.* studied noninvasive *in vivo* pharmacokinetics of Pt drugs in solid tumors. The radioactive Pt drugs were administered in Walker 526 solid tumor bearing rats. Radio-imaging provided a clear picture of accumulation of the drugs and their metabolites in tumors as well as other organs [132]. In a recent study, Vogel and co-workers demonstrated the suitability of ^195m^Pt-labeled Pt compounds including [^195m^Pt]-CDDP for SPECT imaging for *in vivo* biodistribution study with improved accuracy [133]. Leeuwenburgh and co-workers utilized [^195m^Pt] labeling strategy to study bone targetability of a bisphosphonate-functionalized Pt anticancer drug using micro-SPECT/CT imaging in mice [134]. In a few earlier studies, ^191^Pt was also used to radiolabel Pt drugs for studying biodistribution and kinetics in patients undergoing cisplatin treatment [135,136]. However, its high energy γ photons (539 keV) are not ideal for high contrast radioimaging using modern γ-camera [132,133]. Moreover, unlike ^195m^Pt which decays to ^195^Pt, ^191^Pt decays to ^191^Ir which is a different molecule with different pharmacokinetics. Importantly, it was demonstrated that the radioactive Pt drugs showed better tumor retardation as compared to their nonradioactive parent drugs *in vivo* [137,138]. This is due to simultaneous chemo- and radiotherapy effects at the same spot. However, for clinical application, routine production and supply of these theranostics is essential which is difficult because of very high cost of radioactive Pt and logistical problems [132]. The ^13^N radiolabeled cisplatin [^13^N]-CDDP was also designed to study the pharmacokinetics of cisplatin in mice and in brain tumor patients [131,139]. However, its clinical use is limited due to very short half-life of ^13^N (*t*_1/2_ = 10 min).

A slightly different approach was adopted by Lamichhane et al*.*, who appended a radioactive ^18^F nuclide to a carboplatin derivative through a C4 linker to construct the PET theranostic [^18^F]-FCP (Figure 7A) [140]. ^18^F is one of the widely used radionuclide for construction of clinical PET tracers due to its optimum *t*_1/2_ (109.7 min) and well-established diverse chemistry for its incorporation into various molecules [141,142]. It is worthy to note that longer *t*_1/2_ may cause unwanted tissue damage by radiation whereas ultrasmall *t*_1/2_ may cause difficulties in tracer production and image acquisition.

The most common ^18^F-based PET tracer is ^18^F-FDG (2-fluorodeoxyglucose) which is extensively used for cancer diagnosis and treatment monitoring [12,143,144] [^18^F]-FCP exerted comparable antitumor activity of the parent Pt drug carboplatin under *in vitro* conditions in a panel of cell lines. Importantly, presence of the radionuclide offered the opportunity for in-depth understanding of *in vivo* pharmacokinetics of [^18^F]-FCP, which was not possible for the parent drug carboplatin. Dynamic PET imaging of mice treated with this theranostic agent showed a clear picture of *in vivo* excretion profile of the molecule. Biodistribution study revealed major accumulation in the kidneys and liver after 5 min of administration. However, the compound was cleared out efficiently from body within 90 min (Figure 7B). The tracer was then tested for its ability to distinguish between tumor types and assess tumor heterogeneity. [^18^F]-FCP was administered intravenously via tail vein into mice bearing KB-3-1 and COLO-205 xenografts. PET images acquired at 90 min post injection showed obvious differences in uptake of the theranostic between KB-3-1 (2.6% ID/g) and COLO-205 (0.8% ID/g) tumors (Figure 7C). Furthermore, heterogeneity in tracer uptake was evident in KB-3-1 tumor. This study enabled the imaging of intertumoral drug distribution and the detection of heterogeneous retention within the tumor.

As discussed above, more and more Re-complexes are being investigated for their anticancer potential and several potential candidates have already been identified. The congener pair of Re and radioactive ^99m^Tc (*t*_1/2_ of 6.02 h) is an ideal pair for development of SPECT-based theranostics. Both these metals can be interchangeably incorporated within the same organic ligand. Therefore, a Re complex could be used for therapy and the corresponding ^99m^Tc complex could be employed for imaging. Wilson et al*.* identified the rhenium tricarbonyl complex **30** with excellent anticancer properties (Figure 7D) [65]. While the inherent luminescence property was exploited to evaluate cellular uptake and mechanism of anticancer activities *in vitro*, the ^99m^Tc analog of **30** (30*) was synthesized to study the biodistribution and the excretion profile of this class of compounds in C57B16 mice. Instead of imaging, the authors used γ-counter to quantify the residual radioactivity in different organs harvested from mice after 30, 60 and 120 min of 30* administration. Results suggested rapid renal and hepatic clearance without significant accumulation in important organs. A biodistribution study of **30** using ICP-MS to quantify Re yielded similar results, suggesting the suitability of a ^99m^Tc analog for pharmacokinetic studies of Re anticancer compounds. Another example involving Re/^99m^Tc congener pair was reported by Gasser et al. [145]*.* They synthesized a heterobimetallic Pt-Re anticancer agent **31** (Figure 7D) by connecting an unconventional *trans*-chlorido Pt(II) complex to a photoactivatable Re tricarbonyl unit. The complex exhibited lower cytotoxicity in MRC-5 nontumoral cell line as compared with cisplatin and had similar cytotoxicity in A2780 and A2780R cell lines. This pointed to the potential of this class of heterobimetallic molecules to circumvent cisplatin resistance. The cellular uptake of the molecule was studied by using confocal microscopy in HeLa cells. The compound was homogeneously distributed throughout the cell. Subsequent investigations on the mechanism of action suggested that the molecule exerted its cytotoxicity via photosensitization and subsequent ^1^O_2_ generation. To prove the theranostic capability of the complex, the authors synthesized the ^99m^Tc analog (31*). 31* was used to study *in vivo* biodistribution in Balb/C mice using nuclear imaging [145]. The compound primarily accumulated in the excretory organs such as liver and kidney, and had a slow blood clearance due to its lipophilic nature. Further *in vivo* efficacy study using xenograft model needs to be carried out for a better understanding of its anticancer potential.

Moving away from the Re/Tc pair we'll now have a look at a theranostic in which a therapeutically relevant metal center was covalently conjugated to a radioactive entity. The theranostic design by Bodio and coworkers featured a DOTA chelated radioactive ^153^Sm unit bearing a triaryl phosphine coordinated to Ru^II^(arene) anticancer unit (**32**, Figure 7D) [146]. Despite challenging synthesis, the bimetallic complex was obtained with decent yield and high purity. Compound **32** showed poor antiproliferative properties *in vitro*. It is important to note that low IC_50_ values are not observed for RAPTA-type metallodrugs. Nevertheless, *in vivo* biocompatibility studies in healthy CD-1 mice showed that **32** did not show toxicity to the animal and biodistribution was examined after 1 h of administration. Long circulation time in the blood and fast clearance from soft tissues was observed. Majority of the compound accumulated in the kidney, liver and intestine. The *in vivo* anticancer efficacy of this candidate was not evaluated.

## Theranostics comprising IR imaging

In the recent past, organometallic carbonyl compounds with M-CO bond(s) (where M = metal and CO = carbonyl ligand) have found widespread application in biology. A large variety of such complexes of cobalt, iron, chromium and rhenium with excellent photoactivated CO releasing abilities as well as anticancer properties have been reported [147–149]. Interestingly, metal-carbonyls (M-CO) absorb strongly in the 1800–2200 cm^−1^ region, which is free of interference from biomolecules present in biological samples or cells [47,150,151]. This frequency window is commonly known as the ‘mid-IR transparency window of cells’ which offers the possibility of studying metal carbonyls using IR imaging in biological media [47]. In a typical experimental setting, cells are treated with the metal carbonyl probe and the IR mapping of amide-II (1545–1539 cm^−1^) [152] and M-CO bands are recorded using an IR microscope to define the overall shape of the cell and distribution of the metal carbonyl compound respectively [47]. It is worthy to note that studies on employment of IR-imaging to monitor cellular uptake and intracellular localization of metal carbonyls with anticancer properties are rare.

**Figure 8:**
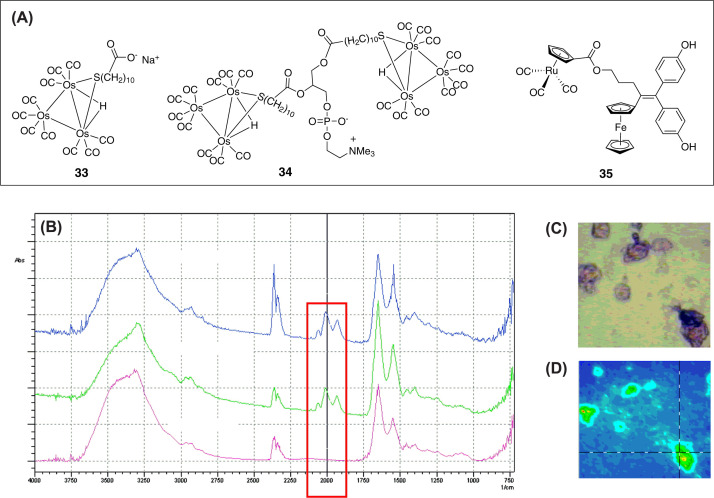
IR-based theranostics (**A**) Structures of osmium-carbonyl conjugated fatty acid **33** and phosphatidylcholine **34**. (**B**) IR spectra of untreated (bottom), **33** treated (middle) and **34** treated (top) HL60 mucosa cells. The Os-CO stretching vibrations are highlighted by red box. (**C**) Optical image of HL60 mucosa cells treated with **33**. False color IR imaging of HL60 mucosa cells treated with **33**. Figure 8b and 8c were reproduced from ref [150]. with permission from American Chemical Society.

To illustrate the concept of IR-based anticancer theranostics, we will discuss a few key examples. The feasibility of IR-imaging of biologically relevant metal carbonyls in cell was first demonstrated by Leong et al*.* in 2007 [150]. HL60 cells were incubated with osmium-carbonyl conjugated fatty acid (**33**, Figure 7A) or a phosphatidylcholine (**34**, Figure 8A) and IR bands were recorded. The distinct band corresponding to M-CO at 2013 cm^−1^ was clearly visible in cells treated with **33** and **34** (Figure 8B). This band is absent in the IR spectrum of untreated cells. The IR-mapping of the amide band-I and M-CO band suggested that the compounds were internalized (Figure 8C,D). However, due to limited spatial resolution, the exact intracellular distribution of these compounds could not be accurately pinpointed. Over the years, various aspects of this technique have been significantly improved. For example, the synchrotron radiation FTIR spectromicroscopy (SR-FTIR SM) allowed imaging of living cells with a spatial resolution in the low micron range [46,48]. Policar, Vessieres, Jaouen and others have successfully applied the SR-FTIR SM technique to image various Re(CO)_3_ compounds in a single cell, albeit information on antiproliferative potencies of these compounds was not available [48,151,153–155].

Inspired by the tamoxifen and ferrocifen class of anticancer agents [155]. Vessieres and co-workers synthesized various CpRe(CO)_3_ tethered derivatives of tamoxifen and ferrocifen (e.g. **35**, Figure 8A) which exhibited excellent antiproliferative activity in triple-negative breast cancer MDA-MB-231 cells [154]. The bimetallic compound **35** exerted antiproliferative activity with an IC_50_ of 2.5 µM. Successful intracellular mapping of **35** in a single MDA-MB-231 cell was done by AFM-IR imaging. Results suggested that the compound readily entered the cells and localized mainly in the nucleus.

## Conclusion and perspectives

The field of ‘theranostics’ has evolved significantly since it was first coined in 2002. Cancer, which is the second leading cause of fatalities worldwide, continues to impose a huge socio-economic burden in the society. Chemotherapy remains the primary treatment modality for a variety of cancers. Despite promising anticancer efficacy of small molecule chemotherapeutic drugs, their side effects compel us to look for alternate, safer treatment options. In this context ‘theranostics’ combining diagnostic and imaging properties are going to play an important role. The ability to simultaneously treat and monitor the therapeutic outcome is going to usher in the concept of ’personalized medicine’ in cancer treatment. Moreover, the opportunity to track a new drug candidate molecule in cells and living organism will speed up its pharmacological evaluation in the developmental stage.

The clinical success of chemotherapeutic Pt drugs cisplatin, carboplatin and oxaliplatin encouraged researchers to investigate anticancer potential of various Pt and non-Pt metal complexes. Several potential metal-based anticancer compounds have been identified and a few have also entered clinical trials. In this review, we highlighted the design principle and biological aspects of different classes of metal-based anticancer theranostics. We discussed key examples of theranostic candidates to emphasize how the possibility of imaging facilitates the evaluation of cellular uptake and intracellular distribution in cells as well as tumor accumulation and pharmacokinetics in animal models. The unique properties of metal centers in terms of their chemistry and cytotoxic activity opens up a plethora of possibilities for future research in this area. While the initial results seem to be encouraging, a lot more research needs to be done to fully exploit the potential of theranostic agents: (a) The emission and excitation wavelengths of many luminescent metal-based theranostics fall in the visible region. This renders them ineffective for imaging deep-seated cancers. This issue can be resolved to some extent by using IR-based theranostics which permit deep tissue penetration. However, the low resolution of this technique makes it less attractive as compared to fluorescence-based imaging. Both these problems (tissue penetration depth and resolution) can be circumvented by employing radionuclide-based theranostics. Here, the possibility of radiation damage to healthy tissues, synthesis, stability and half-life of the radioisotope are the pertinent aspects which need to be addressed. Clearly, the development of an imaging modality with greater tissue penetration depth and high resolution is an important research direction. (b) Most of the theranostics we discussed in this review were non-targeted molecules. The molecules can be made targeted by employing either passive or active targeting in order to enhance the efficacy and efficiency of the treatment. (3) The molecules need to be subjected to in-depth biological studies with respect to the cellular uptake, mode of cytotoxic activity, biological targets, stability under *in vivo* and *in vitro* conditions, metabolic profiling, etc. SAR exploration for these molecules is not very straightforward. Further efforts need to be made in order to understand the ‘link’ between chemical structure and its biological implications, both *in vitro* and *in vivo.* We firmly believe that the field of small-molecule metal-based theranostics is going to blossom in the near future. Inputs must be obtained from diverse disciplines like oncology, pharmacology, clinical research and pharmaceutical industries to translate this class of molecules to the clinic.

## Abbreviations

DCF: 2′,7′-dichlorofluorescein
LD: linear dichroism
MMP: mitochondrial membrane potential
MTD: maximum tolerated dose
PS: photosensitizers
TPPLIM: two-photon phosphorescence lifetime imaging microscopy
TrxR: thioredoxin reductase
